# Dopamine agonist inhibits vascular endothelial growth factor protein production and secretion in granulosa cells

**DOI:** 10.1186/s12958-015-0102-4

**Published:** 2015-09-17

**Authors:** Hortensia Ferrero, Carmen M. García-Pascual, Nuria Pellicer, Carlos Simón, Antonio Pellicer, Raúl Gómez

**Affiliations:** Fundación IVI, C/ Catedrático Agustín Escardino, n°9, Paterna, Valencia, 46980 Spain; Instituto Universitario IVI/ INCLIVA, Valencia, 46015 Spain; Hospital Universitario i Politécnico La Fe, Valencia, 46026 Spain

**Keywords:** OHSS, VEGF, Granulosa cells, Dopamine receptor 2, Dopamine receptor-2 agonist

## Abstract

**Background:**

Dopamine receptor 2 agonists (D2-ags) inhibit vascular endothelial growth factor (VEGF) secretion in luteinized granulosa cells (LGCs) both *in vitro* and *in vivo*. However, the mechanism of D2 regulation of the VEGF/VEGF Receptor 2 (VEGFR-2) pathway remains to be elucidated. We sought to determine the effects of D2 signaling on VEGF transcription and translation in LGCs, with the expectation of identifying potential D2-ag-based therapies for ovarian hyperstimulation syndrome (OHSS).

**Findings:**

LGCs from egg donors were cultured with chorionic gonadotropin (hCG) in the presence of Actinomycin-D (ActD) or Brefeldin-A (BFA) to evaluate the effects of a D2-ag, cabergoline (Cb2), on VEGF secretion. The contribution of the conventional G_i_/G_o_, G_z_ and AKT/β-Arrestin pathways in the VEGF regulation was assessed by adding pertussis toxin (PTX), phorbol 12-myristate 13-acetate (PMA), or wortmannin (WT). While Cb2 inhibited VEGF secretion by interfering with VEGF peptide translation and secretion, inhibition of conventional D2 transduction pathways did not reverse Cb2-mediated inhibition of VEGF secretion.

**Conclusions:**

The effects of D2-ag on VEGF translation and secretion are mediated by D2 signaling pathways that have yet to be described. We found that D2-ag inhibits VEGF secretion at the post-transcriptional level, suggesting that D2-ag treatment should be combined with therapies that inhibit VEGF transcription, such as the employment of LH or GnRH for triggering ovulation, to improve the efficacy of OHSS prevention.

## Findings

Ovarian hyperstimulation syndrome (OHSS) is an iatrogenic complication of ovarian stimulation associated with the use of human chorionic gonadotropin (hCG), is characterized by an increase in vascular permeability (VP) [1]. Vascular endothelial growth factor (VEGF) is an important component in the development of OHSS [2, 3]. Besides, it has been demonstrated that hCG administration increases VEGF mRNA expression in luteinized granulosa cells (LCGs) [4, 5]. Several studies have attempted to avoid an increase in VEGF while employing GnRH agonists to induce ovulation [6, 7]; however, this strategy does not totally avoid OHSS onset [8], leaving a need for treatments that block VEGF/VEGF receptor 2 (VEGFR2) signaling completely.

Several *in vivo* [9, 10] and *in vitro* studies [11] have suggested a role for dopamine in the regulation of the VEGF/VEGFR2 pathway. Interestingly, high levels of dopamine are present in follicular fluid and human ovarian biopsies [12, 13], and dopamine receptors have been found on human granulosa cells (GCs) [14]. Previous studies suggest a role for dopamine receptor agonist (D2-ag) in preventing VP increases [15, 16] by inhibiting VEGF secretion [17, 18]. However, D2-ag has previously been shown to block the onset of early-stage OHSS in 50 % of women at risk for developing the condition, but it was not effective in preventing the late onset form [19]. Understanding the molecular mechanisms involved in D2 regulation of VEGF is critical to elucidating the role of D2-ag in OHSS prevention. Therefore, the goal of this study was to determine the molecular mechanism through which D2-ag inhibits VEGF secretion and to establish which D2 signaling pathways described in D2-expressing cells (Gi/Go, Gz and AKT/β-Arrestin) [20] are involved in the regulation of VEGF secretion.

LGCs were obtained from 24 egg donors [aged 25–30 years, oocytes retrieved = 10–15, estrogen (E2) < 2000 pg/mL, body mass index (BMI) < 30]. Cells were isolated by filtering [21], then washed and cultured as described below in the different studies. Written informed consent was provided by all participants, and the study protocol was approved by ethics committee of IVI Valencia.

### VEGF mRNA is not altered by D2-agonist (Cabergoline)

**Fig. 1:**
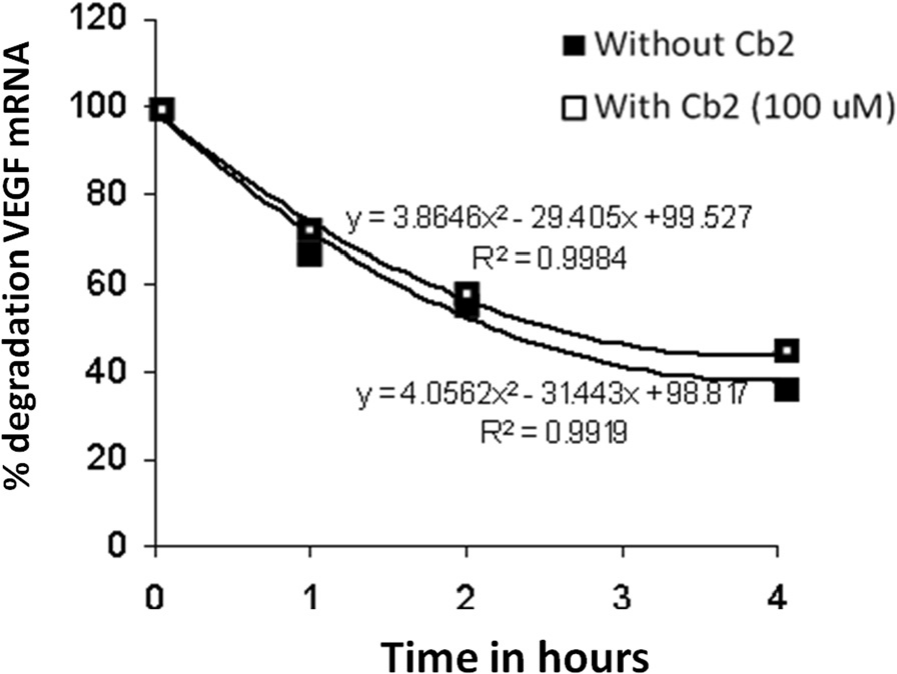
VEGF mRNA is not altered by the dopamine receptor 2 agonist Cabergoline. Time-course experiments designed to study the effects of the dopamine receptor 2 agonist (D2-ag) Cabergoline (Cb2) on VEGF mRNA stability (expressed as the percentage of VEGF mRNA degradation) in LGCs. LGCs (*n* = 6) were pre-incubated with actinomycin D (ActD), then treated with Cb2, hCG, and ActD for 1, 2, or 4 h or untreated. The amount of VEGF cDNA at time point 0 was assigned a value of 100 and the percentage of degraded mRNA was estimated according to variations in the Ct determined by QF-RT-PCR. No significant differences in VEGF mRNA stability were observed in LGCs treated with Cb2 compared to untreated LGCs

Previous studies have suggested that VEGF inhibition by D2-agonist, cabergoline (Cb2), is not exerted at the transcriptional level [14, 15], so we evaluated whether the half-life of VEGF mRNA remained unaffected by D2-ag administration to LGCs. LGCs (*N* = 6) were pre-incubated with 5 μg/mL Actinomycin-D (ActD) (Sigma-Aldrich, St. Louis, MO) for 2 h to inhibit *de novo* transcription. Supernatant was removed and LGCs were incubated in the presence or absence of Cb2 (100 μM; Pharmacia & Upjohn, North Peapack, NJ, USA) with 5 IU/mL hCG (Profasi; Serono Laboratories, Madrid, Spain) and 5 μg/mL ActD for 1, 2, or 4 h. VEGF (Hs00900055_m1) and β-actin (Hs99999903_m1) expression levels were estimated by quantitative QF-RT-PCR using TaqMan® Gene Expression Assays (Applied Biosystems, Carlsbad, CA, USA). No significant differences were observed by a Student’s *t*-test in VEGF mRNA (data not shown) or in transcript stability in Cb2-treated LGCs compared to Cb2-untreated LGCs (Fig. 1). These results support our previous suspicious about Cb2 does not affect at transcriptional level.

### D2-ag interferes with VEGF protein production and secretion

To determine whether Cb2 has translational or post-translational effects on VEGF production, LGCs (*N* = 6) were pre-incubated with a protein secretion inhibitor, Brefeldin-A (BFA; 10 μg/mL) (Sigma-Aldrich, St. Louis, US, A), for 1 h. Subsequently, LGCs were incubated in the presence or absence of Cb2 (100 μM) with 5 IU/mL hCG with or without 10 μg/mL BFA for 8 h. Extracellular and intracellular VEGF levels were measured using ELISA kits (R&D Systems, Minneapolis, US). Intracellular VEGF was confirmed by immunofluorescence using anti-VEGF antibody (10 μg/mL; R&D Systems Minneapolis, MN) and AlexaFluor594 antibody (1:500; R&D Systems Minneapolis, US). A fluorescence microscope was used to observe fluorescence (Nikon Eclipse E400, Japan), and images were acquired with a digital camera (Olympus, Tokyo, Japan). Signal intensity was assessed by Matlab (Worcester, MA), as previously described [22]. The Student’s *t*-test was used for statistical analysis and a *p*-value less than 0.05 was considered statistically significant.

**Fig. 2:**
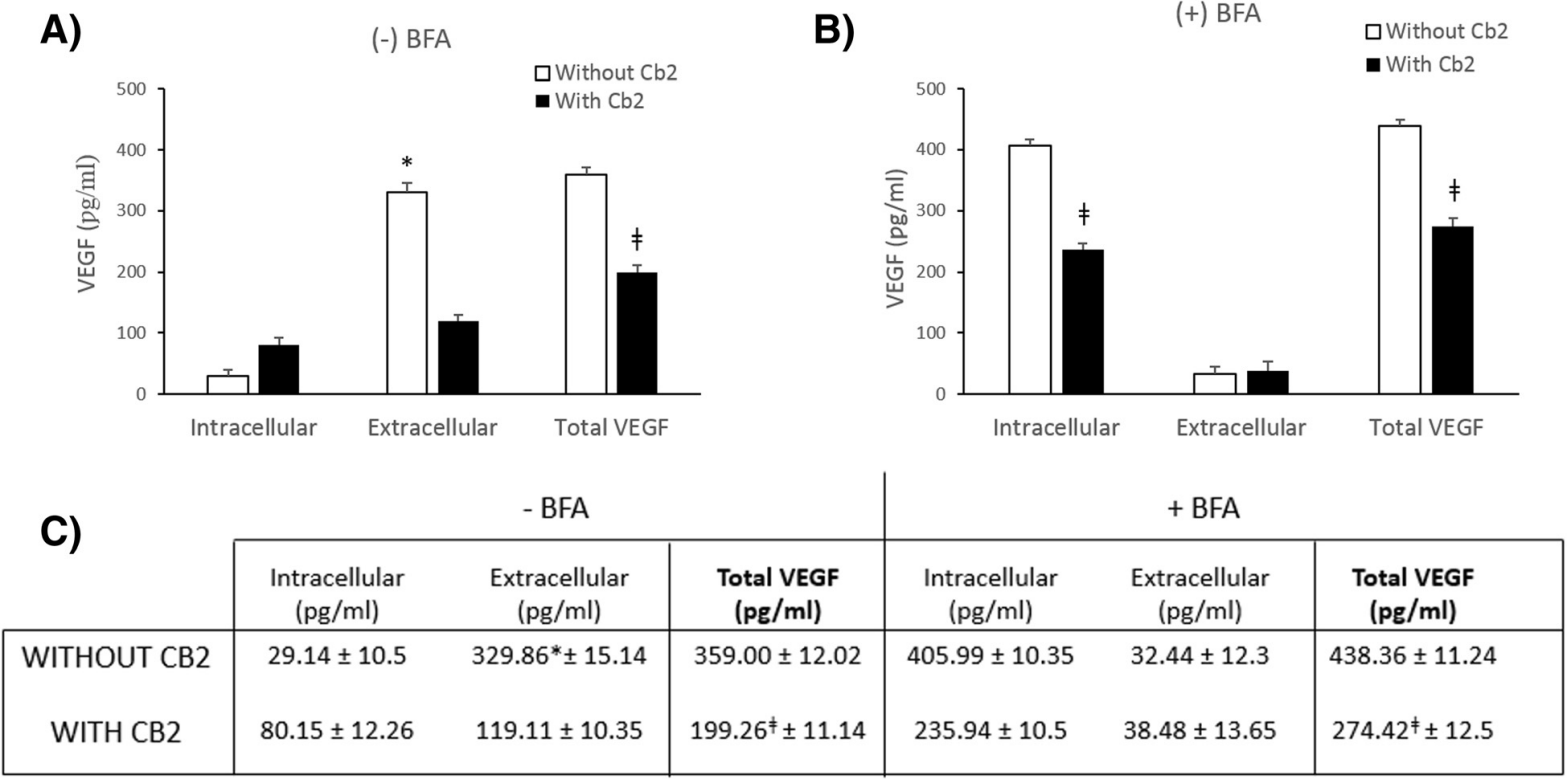
Post-transcriptional regulation of VEGF by the dopamine receptor 2 agonist, Cabergoline. Intracellular and extracellular quantification of VEGF by ELISA in LGCs (*n* = 6) cultured for 8 h in hCG with or without Cb2 in the (**a**) absence or (**b**) presence of BFA. **c** Intracellular, extracellular, and total VEGF levels expressed as pg/mL (*mean ± SD*). In the absence of BFA (−) extracellular VEGF in Cb2-untreated LGCs were higher than intracellular VEGF levels. However, in the presence of BFA (+) the intracellular VEGF in Cb2-treated LGCs is lower than in Cb2-untreated cells. ^ǂ^ *p < 0.05* compared to VEGF levels in Cb2-treated LGCs vs Cb2-untreated LGCs; *p < 0.05 compared to intracellular vs extracellular VEGF levels in absence of BFA

**Fig. 3:**
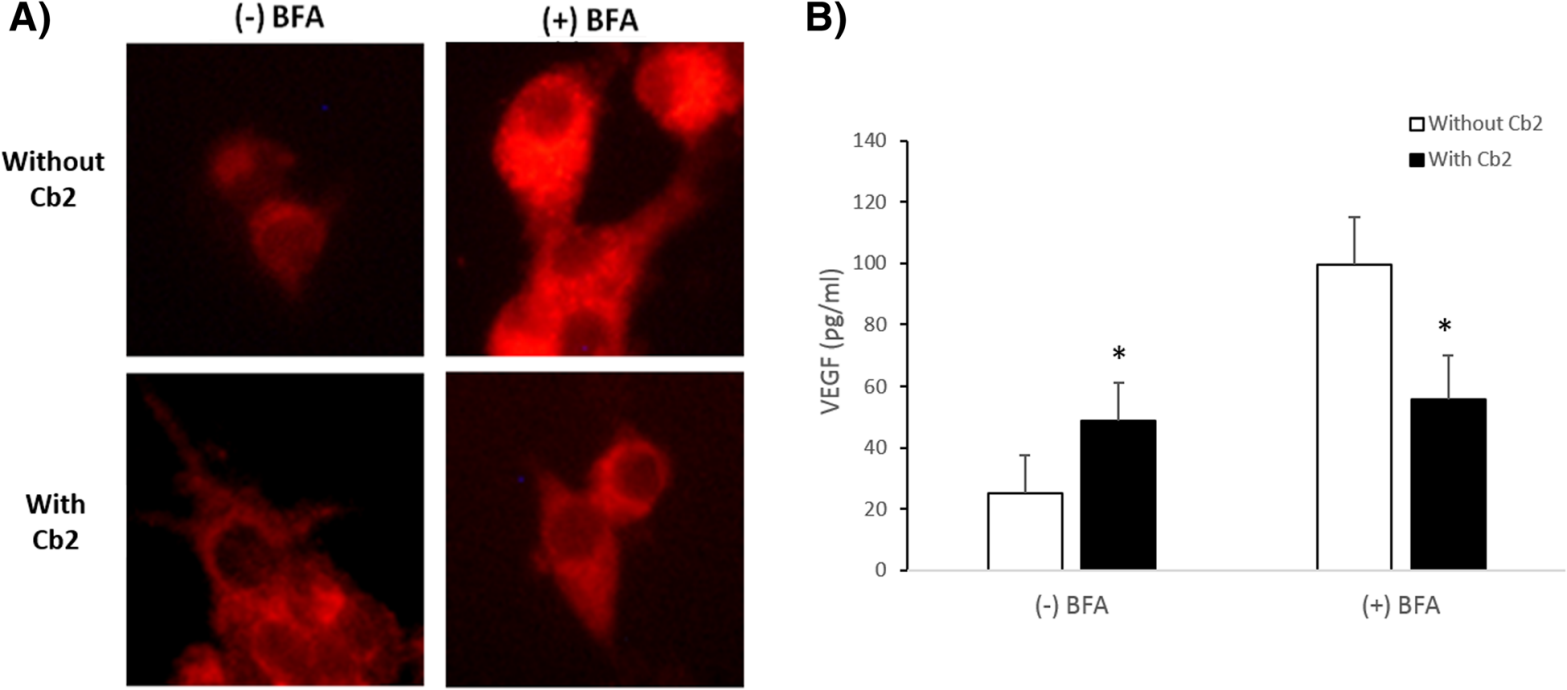
Intracellular VEGF levels in LGCs cultured with the dopamine receptor 2 agonist, Cabergoline. **a** Representative immunofluorescence images of intracellular VEGF staining in fixed and permeabilized LGCs (*n* = 6) pre-incubated with BFA and then incubated in the presence or absence of Cabergoline (Cb2) with hCG plus or minus BFA. **b** VEGF immunofluorescence quantification as assessed by Matlab, expressed as eu/pixel. In the absence of BFA (−), intracellular VEGF levels in Cb2-treated LGCs were higher than the VEGF levels in Cb2-untreated LGCs. However, in the presence of BFA (+) the intracellular VEGF levels in Cb2-treated cells were lower than vehicle-treated cells. *p < 0.05 compared to Cb2-untreated conditions

In the absence of BFA, extracellular VEGF levels in LGCs not treated with Cb2 were higher than intracellular VEGF levels, suggesting that most of the VEGF produced by LGCs were secreted (Fig. 2a, c). However, in Cb2-treated LGCs, extracellular and intracellular VEGF levels were similar. Intracellular VEGF levels in these cells were higher than in Cb2-untreated cells (Figs. 2a, c and 3a, b), suggesting that Cb2 interferes with the secretion of VEGF in LGCs and most of the VEGF produced is not secreted.

When protein secretion was inhibited by BFA, intracellular VEGF levels were lower in Cb2-treated than in untreated LGCs (Figs. 2b, c and 3a, b), suggesting that D2-ag also acts by inhibiting VEGF peptide production. Although there are studies suggesting that VEGF secretion can be regulated by the dopaminergic system [9, 10], none describe the mechanism how D2-agonists inhibit VEGF. In this regard, this is the first study showing that VEGF inhibition by D2-ag is specifically exerted at the post-transcriptional level with effect in of both peptide translation and subsequent releases once it has been produced, suggesting that D2-ag treatment should be combined with therapies to inhibit VEGF transcription, such as LH or GnRH agonists treatment for triggering ovulation, to improve the efficacy of OHSS prevention [6, 7].

### The role of the conventional D2 transduction pathways in the modulation of VEGF secretion mediated by D2-ag

**Fig. 4:**
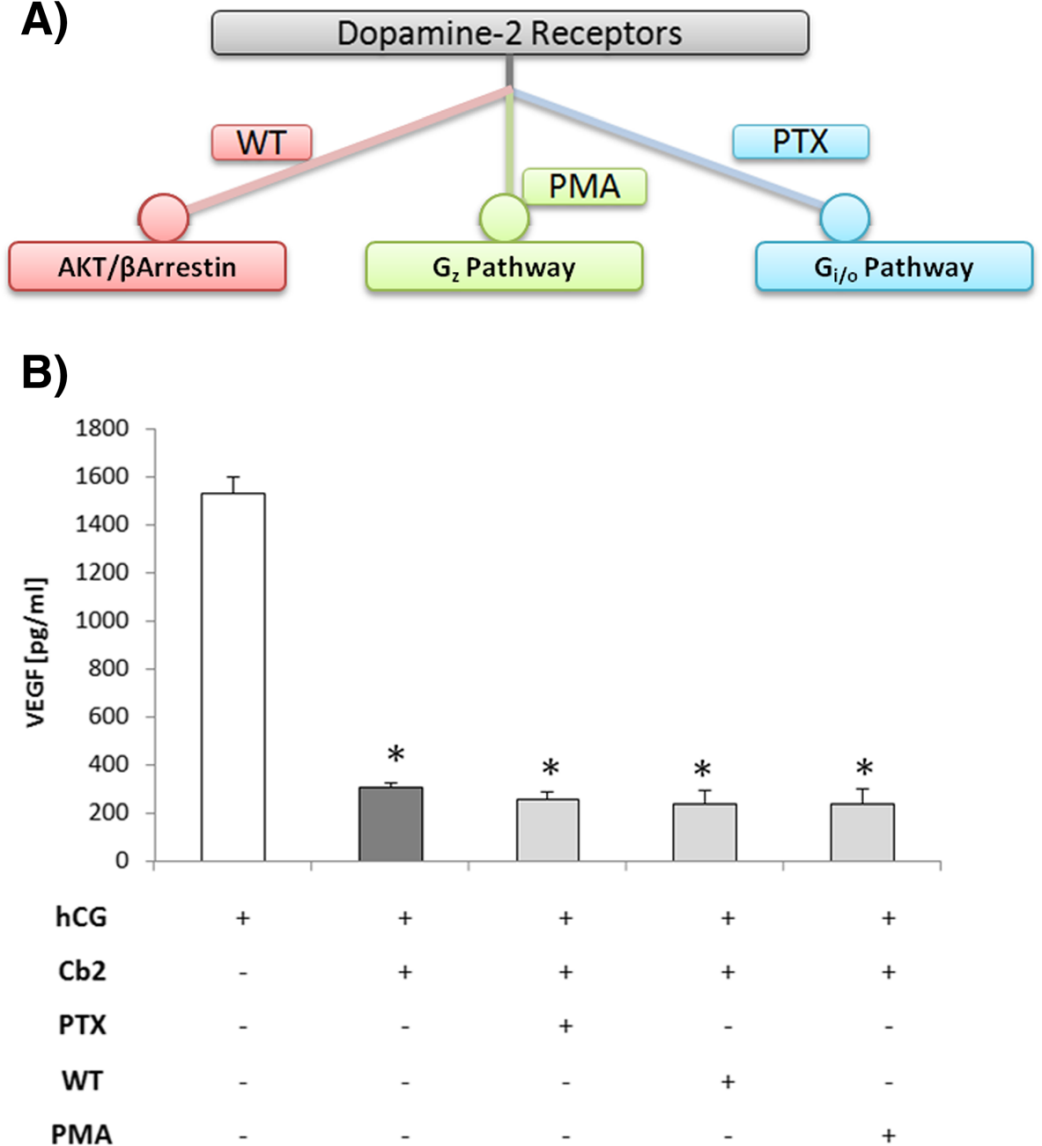
D2-downstream pathway inhibitors do not affect LGC VEGF secretion *in vitro.* **a** Conventional pathways through which D2 signals are transduced and the inhibitors commonly used to block them; PTX = pertussis toxin, WT = wortmannin, and PMA = phorbol 12-myristate 13-acetate (*circles* indicate inhibitory actions). Each of these compounds inhibits one of the D2-downstream pathways. **b** VEGF secretion levels measured by ELISA (expressed as picograms per milliliter) from LGCs (*n* = 6) cultured with hCG in the presence or absence of Cabergoline (Cb2) and PTX, WT, or PMA. Inhibitors of D2-downstream pathways did not reverse the effect of D2-ag on VEGF secretion. *p < 0.01, compared to the (control) D2-ag treated (blank bar) group

To determine whether the conventional D2 transduction pathways (Fig. 4a) described in D2 expressing cells [16] are involved in D2-mediated post-transcriptional VEGF regulation, LGCs (*N* = 6) were incubated with 5 IU/mL hCG in the presence or absence of Cb2 (100 μM) with or without 200 ng/mL pertussis toxin (PTX; a G_i/o_ pathway inhibitor), 100 ng/mL phorbol 12-myristate 13-acetate (PMA; G_z_ pathway inhibitor) or 10 μM wortmannin (WT; a AKT/β-arrestin complex inhibitor) (Sigma-Aldrich, St. Louis, MO, USA) for 48 h. ELISA kit was used to quantify protein production and secretion and a Student’s *t*-test was used to determine statistical significance. Inhibition of these three different pathways failed to counteract the effects of Cb2 on the ability of LGCs to secrete VEGF (Fig. 4b).

Based on our previous findings [17] it is unlikely that the D2 receptor is not involved in the process. Since the Gi/o, Gz, and AKT/β-arrestin signaling pathways are common intermediaries, it is possible that its inhibition might also be acting on pathways other than those transduced by D2, which might be involved in the VEGF regulation in LGCs. Therefore, it could explain why was not observe a counteracting increase in VEGF in response to treatment with PTX, PMA, or WT. The VEGF inhibition observed may be a result of D2-agonist-mediated transduction pathways that have yet to be described or alternative mechanisms that could be acting in well-known VEGF signaling pathways, such as FAK and MAPK pathways [23], PCL/PKC/Sp1 and adenylate cyclase/PKA/CREB pathways [24], or PI3k/AKT pathway [25]. We are unaware whether VEGF regulation by D2-ag is a universal mechanism of angiogenesis control in many types of cells and conditions. If so, our findings, even if non-conclusive, could be useful at least to identify possible candidates involved in the process.

D2-agonists have also been considered for the treatment of tumorigenic conditions, such as ovarian cancer [26], in which the deregulation of the VEGF/VEGFR-2 pathways plays a major role. In this regard, investigation of the mechanism by which VEGF secretion is regulated by the dopaminergic system is needed to provide a basis for more specific therapies to treat diseases related to VEGF-mediated vascular angiogenesis, including OHSS.

Based on our findings, D2-ag in conjunction with clinical strategies aimed at reducing VEGF mRNA levels may provide a more powerful inhibitory effect on VEGF and improve efforts to prevent OHSS.
